# Manufacturing Industry Cancer Risk in Japan: A Multicenter Hospital-Based Case Control Study

**DOI:** 10.31557/APJCP.2020.21.9.2697

**Published:** 2020-09

**Authors:** Rena Kaneko, Yuzuru Sato, Yasuki Kobayashi

**Affiliations:** 1 *Department of Gastroenterology, Kanto Rosai Hospital, 1-1 Kizukisumiyoshi-cho, Nakahara-ku, Kawasaki, Kanagawa, 211-8510 Japan. *; 2 *Department of Public Health, Graduate School of Medicine, The University of Tokyo, 7-3-1 Hongo, Bunkyo-ku, Tokyo, 113-0033 Japan. *

**Keywords:** Manufacturing industry, cancer risk, occupational hazard, case control study

## Abstract

**Background:**

It is well known that specific occupations can cause harm in developing malignant neoplasms. Chemical exposure is particularly high in the manufacturing industry and workers in this sector may face a higher occupational risk for cancer. We aimed to estimate inequalities in the risk of cancers related to occupational chemical exposure in various manufacturing categories.

**Methods:**

Using nationwide clinical inpatient data (1984−2017) in Japan, we undertook a multicenter, case-control study with regard to risks of developing cancers among various manufacturing industry categories. Using the food manufacturing industry as the reference group, odds ratios and 95% confidence intervals for each industry were estimated by conditional logistic regression, adjusted for sex, age, admission period, and the admitting hospital. Medical record summaries accounting for 89% of industrial categories with high odds ratios were collected to confirm diagnoses made on the basis of histology. We estimated industrial hazards based on the Pollutant Release and Transfer Register.

**Results:**

A reduced risk for some of common cancers was observed among lumber and wood products industries. Leather tanning, leather products and fur tended to show a higher risk: 2.36 (95% CI 1.15−4.83) for pancreatic cancer, 2.85 (95% CI 1.26−6.47) for liver cancer and 2.00 (95% CI 1.01–3.99) for lung cancer. For the electronics category, observations of high risk ranged from 2.09 (95%CI 1.18–3.70) for ureter cancer, to 2.49 (95% CI 1.79–3.55) for kidney cancer.

**Conclusions:**

This study revealed industry risk inequalities in manufacturing categories were present with regard to the risk of common cancers in Japan.

## Introduction

Occupations in particular are a major social determinant of health (Marmot, 2005). It is well known that specific occupations can cause harm in that workers may be more susceptible to developing malignant neoplasms.

Previous studies have investigated the association between occupation and cancer mortality and morbidity (Wada et al., 2012; Eguchi et al., 2017; Kaneko et al., 2019). With regard to occupation as an etiology of cancer, occupational exposure to carcinogens can increase the effective risk for cancer (Chen and Osman, 2012; Vlaanderen et al., 2013). In particular, manufacturing processes may involve many occupational exposure to many potentially hazardous carcinogens (Alguacil et al., 2000; Ferris et al., 2013), such as heavy metals (e.g., beryllium, cadmium, mercury, and chromium) and organic solvents (e.g., benzene, chloroform, and phenol), and asbestos(IARC, 1987). The risk may be linked with a combination of other exposures such as smoking or second-hand smoke (Alberg et al., 2013). It is important to account for the characteristics of carcinogen exposure and cigarette smoking in workplaces across occupations when considering the relationship between occupation and cancer (Alberg et al., 2013). However, established risk factors account for a relative small proportion of cancer cases and most causative factors remain ambiguous (Alberg et al., 2013).

In Japan, emissions and exposure to chemical substances are controlled through various regulations(Ministry of Labor Ordinance, 1972a; Ministry of Labor Ordinance, 1972b; Ministry of Economy, 2018). However, a lack of adequate knowledge of these substances and their associated risks makes it difficult to prevent risk exposure. Within the Japanese national workers’ compensation scheme, one’s lifetime occupational history influences cancer risk(Kaneko et al., 2019). For the prevention of disease associated with the workplace and working environment, workplace management to avoid exposure of chemical substances must be promoted by the employer (Mori, 2013). Evaluating the cancer risk for various manufacturing industrial categories is important for gauging the effectiveness of efforts to reduce carcinogen exposure in the workplace. 

Since an occupational hazard is mainly defined as exposure to carcinogens in the manufacturing industry, the aim of this study was to examine the risks of various manufacturing industries in the development of common cancers. 

## Materials and Methods

We obtained an anonymous dataset extracted from the Inpatient Clinico-Occupational Database of the Rosai Hospital Group (ICOD-R), with the permission of the Japan Organization of Occupational Health and Safety (JOHAS), an independent administrative agency in Japan. Rosai Hospitals now comprise 33 hospitals, located from Hokkaido to Kyushu, in both rural and urban areas. The database contains medical chart information overseen by physicians from 1984 to 2017. The diagnoses were coded according to the International Statistical Classification of Diseases and Related Health Problems, 9th Revision (ICD-9) or 10^th^ Revision (ICD-10). The ICOD-R also included a job history (current and past three jobs) as well as smoking and alcohol habits, and a history of lifestyle-related diseases, using interview and questionnaire completed by a patient at the time of admission. Detailed job histories were coded using 3-digit codes of the standardized national classification, the Japan Standard Occupational Classification and Japan Standard Industrial Classification, corresponding to the International Standard Industrial Classification and International Standard Occupational Classification. Written informed consent was obtained before patients completed questionnaires, and trained registrars were in charge of registering the data. The profiles of the inpatients are nationally representative because the Rosai Hospital Group has grown to cover all occupations since the establishment of the Rosai Hospital Group by the Ministry of Labour of Japan in 1949. 

With respect to the selection of cases and controls, we chose 11 common cancer cases defined below that were engaged in the manufacturing industry, and had complete data on birthday, sex, ICD-9, ICD-10 code, history of smoking and alcohol consumption. Cancer sites were selected according to national statistics in Japan (Inoue et al., 2012; National Cancer Center, 2019), with the most common cancer sites being: prostate, breast, kidney, ureter, bladder, esophagus, stomach, liver, pancreas, colon and lung. The prevalence of these cancers was almost identical to that recorded by Japanese national statistics (Zaitsu et al., 2018b; Kaneko et al., 2019).

We randomly sampled one control for each cancer case from cases with fractures of the arms and legs (ICD-9, 810-829 and ICD-10, S40-S99), matched for age, sex, period of admission date, and admitting hospital. Case-control datasets were created for each cancer and were used for subsequent analysis.

 In a case-control dataset, we conducted a multicenter, hospital-based matched case-control study. A conditional logistic regression was conducted for the estimation of ORs, with 95% CIs for each manufacturing industry category in relation to the risk for each cancer. The food manufacturing group was selected as a reference category in accordance with a previous study(Wada et al., 2016; Kaneko et al., 2019). To evaluate the ORs of each industry, we chose the longest-held job of each patient from their industrial history according to previous studies(Zaitsu et al., 2018b; Kaneko et al., 2019). Manufacturing industry categories were classified into 24 categories according to the middle classification of the Japan Standard Industrial Classification of 2013. The 24 manufacturing industry categories were as follows: foods; beverages, tobacco and feed; textile mill products; clothes and other textiles; lumber and wood products, except furniture; furniture and fixtures; pulp, paper and paper products; printing and allied industries; chemical and allied products; petroleum and coal products; plastic products, except otherwise classified; rubber products; leather tanning, leather products and fur; ceramic, stone and clay products; iron and steel; non-ferrous metals and products; fabricated metal products; general-purpose machinery; electrical machinery, equipment and supplies; information and communication electronics; electronic parts, devices and electronic circuits; transportation equipment; precision machinery; and miscellaneous manufacturing industries.

Age was categorized every 5 years. The period of admission was categorized into four study periods (1/1/1984–12/31/1990, 1/1/1991–12/31/2000, 1/1/2001–12/31/2010, 1/1/2011–12/31/2017) and matched into pairs. The regression models included age, sex, period of admission, and admission hospital as factors. Smoking (Brinkman Index), and consumption of alcohol were included as covariates. The Brinkman Index was calculated as the number of cigarettes smoked per day multiplied by the number of years spent smoking cigarettes.

All p-values were two-sided, and p <0.05 was considered statistically significant. All analyses were conducted using STATA/MP15.0 (Stata Corp LP, College Station, TX, USA).

In speculating on industrial chemical hazards becoming carcinogenic, we used data from the Pollutant Release and Transfer Register (PRTR)(Ministry of Economy, 2018). The PRTR is regulated by the Japan Chemical Substance Control Law and enabled us to obtain detailed data of chemical substances used by business operators.

An additional data collection was conducted on all cases for which the record of histological diagnoses was available. Although the ICOD-R database was constructed from medical chart information, indicating that all the diagnoses were clinically accurate, histological diagnoses further ensured a rigorous proof of cancer. We collected medical records for target cases with a high odds ratio for various industrial categories in combination with cancer. Because manpower at each facility was not uniform due to differences in hospital size and location, there was limited ability to collect summarized medical chart. Consequently, we abandoned the collection of low odds ratio combinations and used only high odds ratio combinations. 

The study was approved by the ethics committees of The University of Tokyo (No.10891) and Kanto Rosai Hospital (No.2018-11).

## Results

**Figure1:**
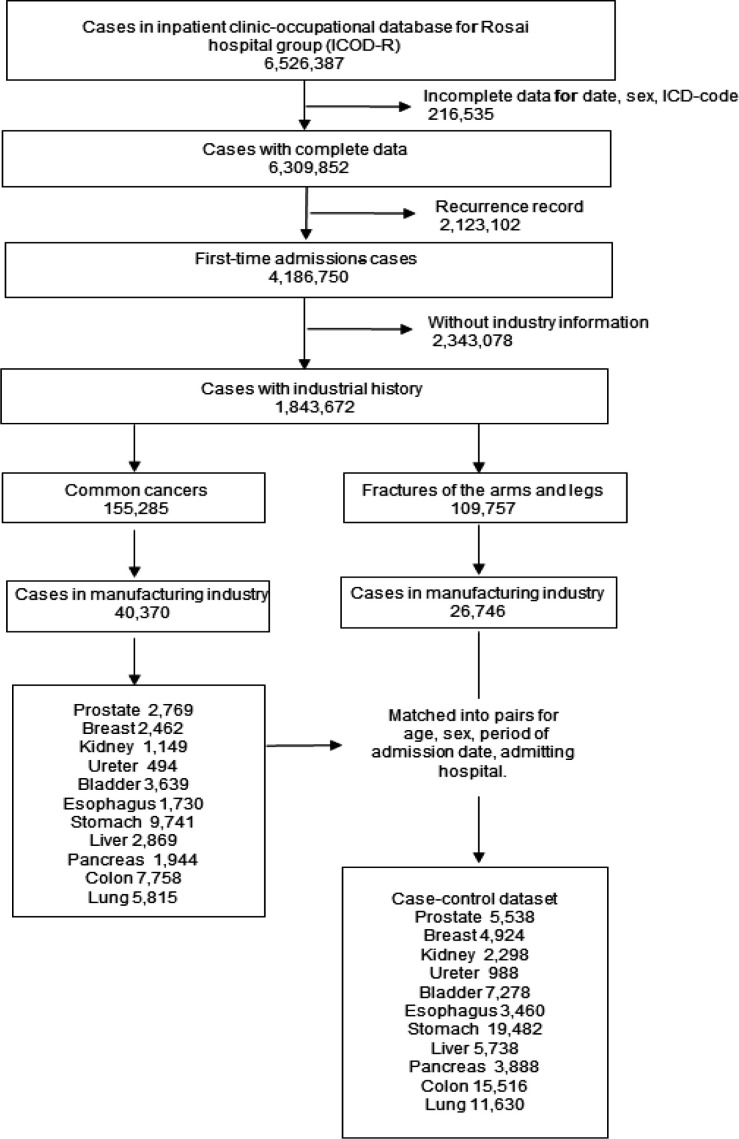
The Data Extraction Flowchart

The data extraction flowchart is shown in Figure 1. The total number of inpatient cases registered in ICOD-R from 1984 to 2017 comprised 6,526,387. Of these, completed data was available for 6,309,852 cases, which included birthday, sex, ICD-9 or ICD-10 code, history of smoking and alcohol consumption. Of these cases, 4,186,750 were first-time admissions cases, while industry information was available for 1,843,672 cases. The common cancers of prostate, breast, kidney, ureter, bladder, esophagus, stomach, liver, pancreas, colon and lung applied to 155,285 cases. Of these, manufacturing industry employees were represented by 40,370 cases. There were 26,746 fracture cases engaged in the manufacturing industry and control was extracted. Therefore, the case-control data set for each cancer was double the number of each cancer case.

The demographics of each cancer and control cases are shown in Table 1.

The onset of breast cancer was the lowest of all cancer sites (55.5 ± 12.1 years). Other cancers showed an onset at around almost 65 years of age. The age of onset of kidney cancer for plastic products, precision machinery, and electronic-related manufacturing industries (electrical machinery, equipment and supplies, information and communication electronics, electronic parts, and devices and electronic circuits) were under 60 years of age (56.8 ± 8.8, 55.2 ± 9.6, 59.3 ± 12.9, 55.1 ± 11.7, and 56.8 ± 10.7). The mean age of onset of cancer for those engaged in electronic parts, devices and electronic circuits were lower than that of other categories for kidney, stomach and colon cancers (56.8 ± 10.7, 56.6 ± 11.6, and 58.6 ± 11.9 years). Overall, there were roughly 4 times as many males (32,238) as females (8,132). About 30% of female cases were breast cancer cases, reflection the low employment rate of women in Japan. The male-female ratios for all types of cancers were consistent with Japanese official data(Ministry of Health, 2020).

Table 2 shows the Brinkman Index and amount of alcohol consumed for each manufacturing category for all cancers and the control group. Both the Brinkman Index and alcohol consumption were heavily biased in cancer cases. It is likely that heavy smokers within an industry category also had a tendency for a high degree of alcohol consumption.

Table 3 shows the ORs for each manufacturing category. For lumber and wood products except for furniture, the ORs for prostate, bladder, esophagus, stomach, liver, pancreatic, and colon cancers were low, ranging from 0.51 (95% CI 0.38–0.67) for liver cancer to 0.74 (95% CI 0.61–0.88) for colon cancer. In the fabricated metal products category there were also some cancer categories that had ORs less than 1.0 and were statistically significant: i.e., 0.76 (95%CI 0.61-0.94) for prostate cancer to 0.83 (95%CI 0.71-0.96) for lung cancer. 

Some categories were over 2.0 times the OR. The OR for ureter cancer was 2.82 (95% CI 1.19–6.70) for rubber products and was 2.01 (95% CI 1.15–3.52) for printing and allied industries. For leather tanning, leather products and fur, the OR for liver cancer was 2.36 (95% CI 1.15–4.83), for pancreatic cancer it was 2.85 (95% CI 1.26–6.47) and for lung cancer it was 2.00 (95% CI 1.01–3.99). For electrical machinery, equipment and supplies, the OR for kidney cancer was 2.49 (95% CI 1.75–3.55), and for ureter cancer it was 2.09 (95% CI 1.18–3.70). For information and communication electronics, the OR for kidney cancer was 2.69 (95% CI 1.77–4.11), and for ureter cancer it was 2.14 (95% CI 1.02–4.45).

Among 106 cases in the leather tanning, leather products and fur category, medical records were collected for 44 cases from all hospitals. Among the latter cases, 30 cases of histological diagnosis of cancer were confirmed. Medical records were not available for the remaining 62 cases because they had not been retained (the legal storage period for medical records in Japan is 5 years). Among cases corresponding to an OR >2.0 in Table 3, there were 134 kidney cancer cases in electrical machinery, equipment and supplies; information and communication electronics; and electronic parts, devices and electronic circuits categories; medical records of 77 cases were available and records for 57 cases had been discarded. A histological diagnosis of cancer was confirmed in 76 cases. Of the 61 ureter cancer cases in printing and allied industries; rubber products; electrical machinery, equipment and supplies; and information and communication electronics; medical records for 34 cases were available and records for 27 cases had been discarded. Of the 34 cases with records, 33 cases were confirmed with histological diagnosis.

In total, cases with histological diagnosis confirmation comprised 89.7% of all cases with available medical records.

## Discussion

This is the first investigation of the inequalities among manufacturing industries in Japan in relation to the risks of common cancers within the country. This study demonstrated a low cancer risk for lumber and wood products; a high cancer risk for rubber products, leather tanning, leather products, and fur, electrical machinery, equipment and supplies and information and communication electronics, in some cancers.

Japanese workers work so long and hard, occupational chemical exposure may strongly influence cancer incidence in Japan (Kaneko et al., 2019). Employees working in manufacturing conditions have a greater potential to genotoxic overexposure than those in other working categories (Choi et al., 2018).

Like benzene glue poisoning in the “Hep sandals shoes case” and the opportunity to establish an “ordinance on the prevention of organic solvent poisoning” in Japan, regulatory laws have always been instituted following the occurrence of a serious manufacturing incident. Because developing cancer caused by occupational exposure requires long incubation times and rare cases are only noticed after workplace aggregation, it is difficult to detect occupational hazards and prevent adverse events beforehand. Therefore, such exploratory and watchful studies like this are important especially on particular industrial categories with occupational chemical hazards.

Concerning the manufacture of wood products, industries associated with exposure to wood dust were deemed as having carcinogenic factors in International Agency for Research on Cancer (IARC) monographs(IARC, 1995): i.e., furniture and cabinet-making were designated as carcinogenic to humans (Group 1), and carpentry and joinery as possibly carcinogenic (Group 2B). Wood dust has been observed to be a risk factor for cancer in the pharynx, larynx, and nasal and oral cavities. Stomach cancer has also been reported to be associated with exposure to wood dust (Beigzadeh et al., 2019). Exposure of wood product industry workers to formaldehyde is inevitable as the compound is indispensable as an adhesive in the manufacturing of plywood(IARC, 1995; IARC, 2006). Formaldehyde is defined as carcinogen to humans(IARC, 2006). Based on the PRTR, formaldehyde emission amounted to 39,220 kg/year in the wood product manufactural industry, the third highest after chemical manufacturing and transportation equipment manufacturing, while there was no registration for food manufacturing(Ministry of Economy, 2018). The emission of dioxins and dioxin-like compounds into the atmosphere is clearly higher than in other industries except for food manufacturing (wood product manufacturing, 302.8970 mg-TEQ/year vs food manufacturing, 741.4960mg-TEQ/year)(Ministry of Economy, 2018). There have been reports that the most toxic dioxin (2,3,7,8-tetrachlorodibenzo-p-dioxin:TCDD) is statistically associated with cancer mortality (pooled standard mortality ratio = 1.09, 95%CI = 1.01-1.19) (Xu et al., 2016). However, there are no relevant data regarding the relationship between ambient dioxin exposure and cancer development (Xu et al., 2016; Danjou et al., 2019). As far as we know, a reduction in cancer risk, as shown in the present study has not been associated with wood products. Because there is no significant risk reductive factor in characteristics in terms of smoking habits, drinking habits, or age compared with other manufacturing industries, another reason may influential for obtaining a lower risk. It is difficult to confirm the risk of confounding hazardous chemicals, since the current occupational exposure levels in wood product manufacturing seem to be trivial if present at all. This is likely due to proper exposure prevention measures within this industry category. 

Among poor nations, increased risks for a number of cancers, such as pancreatic, skin, kidney, prostate and bladder cancers, have been reported among workers in the tanning industry (Hussain and Sullivan, 2013). In such developing countries, occupational hazards are often not adequately controlled due to insufficient occupational health services (Febriana et al., 2012).

In the present study, an elevated risk of cancers of the digestive and respiratory systems rather than of the urinary system is reported in the category of leather tanning, leather products and fur. In Japan, leather tanning processes have been a traditional industry since ancient times (Ingle et al., 2011). The same tendency has been reported for Italy, (Iaia et al., 2006) and high mortality of pancreatic cancer in leather industry retrospective cohort was reported in Belarus (Veyalkin and Gerein, 2006), a country famous for expert craftmanship in the leather industry. Tanning is carried out using natural or synthetic tannins. The process requires chromate salts, hexavalent chrome salts, arsenic, organic solvents (formaldehyde, butyl acetate, ethanol, benzene, toluene), and other toxic compounds. Workers therefore have a high risk of exposure to metal salts (mainly chromates)(Hussain and Sullivan, 2013). IARC classified the tannery processes as “not classifiable as to carcinogenicity to humans”(IARC, 1987). However, the processes involve some chemicals that are classified as “probably carcinogenic” or “carcinogenic to humans”(IARC, 2006). According to PRTR, the amount of chromium and trivalent chromium waste in tanning was 22,770 kg/year, one of the highest levels among manufacturing industries. Metabolized chromium distributes to various organs, causing abnormalities of the kidney, liver, stomach and lung systems (Khan et al., 2013). The extent to which these chemicals may have affected cancer risk in this study is unknown.

To improve pollution management in tanneries, it should be noted that small-scale leather tanneries are superiority to traditional tanning businesses in this regard. In Japan, tanneries are mostly composed of small businesses and small factories, where safety knowledge, safety devices, and work environment management tend to be insufficient, leading the possibilities of exposure to high concentrations of harmful substances among tannery workers. Further efforts to improve work environment management might be necessary.

The manufacture of electrical products is accompanied by various occupational exposures even though each workplace consists of highly controlled clean rooms. Examples of toxic materials include photoactive chemicals, solvents, acids, and toxic gases. Chlorinated solvents used in electrical manufacturing plants have been suggested to be carcinogenic (Oddone et al., 2014). Renal and breast cancer risk in electrical manufacturing have also been reported in the United States and Italy (Karami et al., 2012; Oddone et al., 2014). Together with the rapid increase in the production of semiconductors, the increase in the variety of chemicals with undefined toxicities and corrosiveness requires close attention. 

The strengths of this study include accurate diagnoses that were directly extracted from medical charts, in contrast to the less accurate diagnoses from claims data (Sato et al., 2015). We obtained mostly histological certificates to support the results of analyses. Such micro data has strengthened the results obtained from big data. 

However, several limitations may exist. First, because the ICOD-R was not a relevant population-based database, the dataset may have had a selection bias. In addition, one-third of the missing information within an industry may amplify any selection bias even though all available factors were included in statistics. Due to the missing data, the power of logistic regression may be insufficient for some rare industrial categories. This problem arises because the return of job data is not enforced among patients to protect their privacy. This further adds to selection bias. This point was confirmed in a previous study showing that the occupational profiles used in this database are nationally representative (Zaitsu et al., 2018b).

Second, our study was designed to enable us to assess specific industry exposures related to cancers. Other relevant factors, such as pathogenic organisms (i.e., Helicobacter pylori in stomach cancer, hepatitis virus in liver cancer) or socioeconomic status i.e., amount of income, educational attainment, could not be evaluated due to the limitations of the data. Although our findings did not elucidate a specific relationship between industrial exposure and cancer, the associations identified in this study may be implied.

Finally, evaluating industrial risk by using data from the longest-held job may lead to bias. The occupations used in this study were necessarily the occupations with which individuals were mainly engaged in throughout their lifetime. On this point, a more accurate assessment of industry risk is made than choosing the job of the deceased at the time of death (Eguchi et al., 2017; Tanaka et al., 2017). This might be a lower possibility of misclassification of occupational category compared with the current or most recent jobs (Zaitsu et al., 2018a; Zaitsu et al., 2019a; Zaitsu et al., 2019b). However, this may not always be the most relevant factor for cancer risk. Considering the incubation time from exposure in an industry as a risk of carcinogenicity, future investigations must be made using jobs over a whole lifetime and with consideration of the time lag of developing cancers.

Further detailed studies in future will evaluate the occupational aspects of causal cancer relationships in a more statistical manner.

In conclusion, we have documented industry inequalities in the risk of various cancers in Japanese manufacturing industries. Explicit strategies for industrial exposures are required that take into account industrial activity for the prevention of cancer. On the basis of the information gathered, best management practices in the workplace are recommended for every industrial unit to improve safety for their occupational workers.

**Table 1:** Sex and Age Distribution of All Cases and Manufacturing Categories

	All cancers	Prostate	Breast	Kidney	Ureter	Bladder	Esophagus	Stomach	Liver	Pancreas	Colon	Lung	Fracture(control)
All manufacturing categories n=	40,370	2,769	2,462	1,149	494	3,639	1,730	9,741	2,869	1,944	7,758	5,815	26,746
(male:female)	32,238:8,132	2769:0	49:2,413	978:171	430:64	3,376:263	1,612:118	7,989:1,752	2,518:351	1,535:409	5,991:1,767	4,991:824	20,006:6740
age(mean±SD)	65.3±11.4	68.8±9.0	55.5±12.1	62.5±11.5	69.4±9.9	68.5±11.2	66.4±9.6	64.1±11.7	64.2±10.5	66.4±10.6	65.6±11.1	67.5±10.5	50.8±17.9
Industrial category													
Food n=3570 (male:female)	1,856:1,714	165:0	7:464	44:33	26:7	196:61	121:28	457:375	164:81	98:98	317:378	262:188	1,252:1,459
age(mean±SD)	63.6±11.5	69.6±8.56	56.3±11.1	61.1±11.9	68.9±10.5	68.2±11.7	65.3±8.9	62.7±11.4	63.5±10.6	65.3±9.9	64.9±11.1	64.9±11.2	53.5±18.3
Beverages, tobacco and feed n=612 (male:female)	474:138	52:0	3:40	11:4	7:3	50:3	35:0	110:27	39:8	33:3	81:34	53:16	251:111
age(mean±SD)	65.9±10.8	70.8±9.4	55.7±12.5	63.8±12.8	69.3±6.4	67.6±11.6	68.1±6.4	63.8±11.2	66.7±8.2	67.3±9.8	65.9±10.0	68.6±9.8	52.9±18.2
Textile mill products n=1022 (male:female)	729:293	62:0	1:74	27:5	15:2	80:11	40:7	175:65	71:16	38:19	106:69	114:25	267:210
age(mean±SD)	68.0±11.4	71.6±7.91	59.9±12.1	65.1±8.5	70.3±7.9	72.6±11.3	68.3±9.5	67.6±11.6	63.9±11.1	69.1±12.3	68.3±11.3	70.2±10.6	58.3±19.7
Clothes and other textiles n=1721 (male:female)	595:1,126	50:0	0:340	20:19	6:15	67:28	27:13	160:228	50:55	37:50	116:247	64:129	248:878
age(mean±SD)	64.1±11.8	68.5±11.2	57.3±11.5	65.4±11.7	72.3±10.2	67.5±12.9	67±11.4	64.2±11.6	65.4±9.8	65.5±11.9	66.2±10.9	65.9±10.5	58.8±16.8
Lumber and wood products, except furniture n=1319 (male:female)	1,129:190	94:0	2:49	35:5	16:4	112:8	57:2	299:39	74:8	55:10	186:46	199:19	761:190
age(mean±SD)	67.1±11.5	70.4±10.3	56.2±12.6	65.7±13.5	71.8±8.12	69.8±11.6	66.4±10.9	65.7±11.1	65.6±9.1	69.6±9.5	67.1±11.8	68.9±11.3	54.1±18.3
Furniture and fixtures n=652 (male:female)	561:91	63:0	1:25	17:6	11:1	57:4	26:0	131:23	39:2	19:3	111:23	86:4	314:58
age(mean±SD)	66.2±11.1	70.7±9.7	57.7±11.4	60.4±8.3	74.5±7.82	68.8±9.5	66.7±8.5	64.7±12.5	64.4±12.5	67.2±10.7	65.6±10.5	68.4±10.0	53.5±187.5
Pulp, paper and paper products n=1065 (male:female)	871:194	90:0	2:54	28:4	6:1	84:8	51:2	217:44	58:14	39:12	169:37	127:18	565:166
age(mean±SD)	74.8±11.2	67.0±9.8	58.1±12.4	62.5±11.1	68.1±10.2	66.9±10.5	67.5±10.1	63.6±11.8	62.3±11.2	64.6±10.7	64.6±10.8	67.4±10.0	49.9±19.5
Printing and allied industries n=1316 (male:female)	1,067:249	84:0	0:94	35:6	23:0	130:5	80:2	233:44	82:13	51:15	213:55	136:15	579:215
age(mean±SD)	65.2±11.8	67.8±9.2	54.5±13.4	60.5±11.1	72.0±9.2	67.3±12.0	67.1±9.4	64.8±11.7	66.9±10.5	67.1±10.5	64.5±11.5	67.1±11.0	48.9±18.0
Chemicals, and chemical and allied products n=3656 (male:female)	3,299:357	350:0	0:143	102:11	38:0	336:11	121:28	797:75	233:17	151:9	614:63	532:25	1,634:328
age(mean±SD)	66.5±11.4	69.9±9.4	52.4±12.2	63.1±11.3	70.6±9.1	68.4±11.2	65.2±8.92	63.9±8.2	64.2±8.8	67.5±9.6	66.4±11.6	68.9±10.2	51.5±18.9
Petroleum and coal products n=360 (male:female)	339:21	33:0	0:7	12:0	3;0	36:2	19:0	82:5	20:3	16:0	68:3	50:1	147:17
age(mean±SD)	63.7±11.4	67.7±8.51	45.6±13.2	62.4±9,6	75.6±9.3	65.9±11.2	66.7±10.3	61.5±11.8	68.0±10.5	58.0±15.9	63.4±10.9	64.9±8.6	50.7±16.6
Plastic products, except otherwise classified n=599 (male:female)	421:178	28:0	1:62	16:4	4:1	34:7	32:3	95:38	30:1	19:5	92:38	71:18	377:138
age(mean±SD)	62.4±11.1	63.8±7.8	58.2±10.7	56.8±8.8	67.8±8.5	65.9±12.3	61.5±10.8	61.0±11.6	60.8±11.9	61.7±9.7	64.5±11.7	64.4±9.5	48.7±16.6
Rubber products n=316 (male:female)	248:68	18:0	0:22	8:3	6:1	35:0	14:1	58:14	25:3	11:6	40:14	33:4	136:51
age(mean±SD)	64.7±11.8	71.3±9.3	54.3±12.9	64.2±12.4	60.4±11.3	69.5±10.3	61.3±12.1	63.1±11.3	61.6±10.5	66.8±10.1	66.6±11.9	67.5±10.9	49.5±19.8
Leather tanning, leather products and fur n=106 (male:female)	75:31	5:0	0:10	2:1	1:0	8:0	1:0	23:6	11:1	4:4	11:4	9:5	24:21
age(mean±SD)	61.3±11.1	69.6±9.1	48.8±11.4	70.6±4.2	-	64.3±14.7	-	60.1±11.4	64.9±7.3	59.8±10.0	62.4±9.5	62.5±9.8	50.3±19.9
Ceramic, stone and clay products n=2167 (male:female)	1,878:289	139:0	4:72	45:4	28:1	196:14	88:1	441:70	159:23	77:14	323:56	378:34	1,071:244
age(mean±SD)	66.5±10.9	69.8±8.7	58.2±13.5	63.5±11.5	65.9±11.1	70.0±10.4	66.9±9.5	64.5±11.6	64.9±11.9	67.4±11.4	67.1±10.4	67.9±9.4	52.6±16.8
Iron and steel n=3970 (male:female)	3,746:224	348:0	6:70	106:2	48:0	393:7	173:2	946:46	321:12	179:17	659:42	568:25	1,996:193
age(mean±SD)	66.1±10.6	69.1±8.7	54.0±11.1	64.5±10.4	68.1±9.0	68.9±10.1	65.7±9.5	63.6±11.4	64.3±9.94	66.8±10.5	66.4±10.4	69.0±9.37	50.9±17.7
	All cancers	Prostate	Breast	Kidney	Ureter	Bladder	Esophagus	Stomach	Liver	Pancreas	Colon	Lung	Fracture(control)
Non-ferrous metals and products n=1091 (male:female)	990:101	84:0	3:38	29:2	13:0	112:4	52:2	236:24	65:4	53:3	176:18	168:5	554:59
age(mean±SD)	66.3±10.9	68.9±8.25	55.6±11.7	64.8±11.1	60.3±10.1	68.1±11.0	66.1±10.6	65.1±11.9	64.2±8.8	67.4±9.8	65.5±10.6	69.5±10.0	49.4±17.2
Fabricated metal products n=4614 (male:female)	3,823:791	279:0	3:192	93:13	36:8	342:29	223:9	1,015:193	307:31	178:44	750:203	598:68	2,871:728
age(mean±SD)	66.1±10.6	67.5±8.4	58.0±11.9	63.9±10.6	72.3±10.5	69.1±9.9	65.8±8.9	65.0±8.5	64.9±10.2	67.8±9.3	66.4±10.1	68.3±9.75	51.0±17.5
General purpose machinery n=3992 (male:female)	3,546:446	306:0	6:149	122:8	51:2	377:14	156:4	889:90	281:12	152:23	682:97	524:47	2,328:403
age(mean±SD)	65.5±11.4	69.3±7.8	55.1±13.3	64.2±11.9	68.6±10.9	68.3±10.8	66.4±8.3	64.3±11.7	64.3±10.2	66.9±10.3	64.8±11.4	67.9±11.0	49.3±17.3
Electrical machinery, equipment and supplies n=1497 (male:female)	1,153:344	98:0	2:100	51:12	19:2	125:10	55:3	262:81	65:14	49:13	258:66	169:43	663:241
age(mean±SD)	62.7±12.2	64.1±8.4	49.4±12.1	59.3±12.9	63.5±11.1	68.7±12.4	66.2±10.5	61.9±11.9	60.7±11.8	63.7±10.3	63.8±11.7	64.9±11.3	46.6±16.9
Information and communication electronics n=783 (male:female)	585:198	58:0	1:77	28:7	9:1	65:4	29:3	119:39	28:8	38:10	129:22	81:27	455:151
age(mean±SD)	61.4±12.7	66.6±9.53	51.1±9.7	55.1±11.7	67.9±8.9	61.7±15.3	65±9.3	60.6±12.7	62.4±11.6	61.5±12.4	62.8±12.8	65.7±11.1	42.2±14.9
Electronic parts, devices and electronic circuits n=837 (male:female)	521:316	37:0	0:104	27:9	9:2	60:6	20:1	116:69	31:10	33:18	120:71	68:26	652:267
age(mean±SD)	58.8±11.9	62.6±8.9	50.7±11.3	56.8±10.7	68.9±6.3	64.1±11.5	64.6±14.5	56.6±11.6	61.5±11.1	61.7±9.1	58.6±11.9	62.7±10.6	42.3±14.8
Transportation equipment n=3666 (male:female)	3,306:362	251:0	5:103	91:5	45:8	368:14	140:6	852:58	281:7	160:20	550:96	562:44	2,225:313
age(mean±SD)	66.2±11.2	68.9±8.7	54.1±11.8	62.8±10.8	69.5±10.1	69.5±10.8	66.9±9.2	65.2±11.7	64.9±10.0	67.1±11.2	66.0±11.2	67.33±10.5	48.7±17.8
Precision machinery n=487 (male:female)	386:101	29:0	2:41	12:0	1:0	41:2	21:0	96:19	38:2	21:2	76:25	49:10	279:84
age(mean±SD)	62.2±11.5	65.5±8.8	54.3±12.5	55.2±9.5	-	65.6±10.0	68.9±12.2	60.4±13.3	62.5±8.2	64.1±12.6	62.7±10.0	64.4±9.9	46.2±17.2
Miscellaneous manufacturing industries n=952 (male:female)	659:293	46:0	0:83	17:8	9:5	72:11	31:1	180:80	46:6	24:11	144:60	90:28	357:215
age(mean±SD)	66.0±11.7	71.7±10.7	56.5±13.1	61.1±14.6	72.3±6.3	68.6±11.4	6.9±9.4	66.9±11.2	66.9±11.2	67.3±12.5	66.4±10.4	68:9±10.7	54.8±19.3

In columns that included only one case, age was not disclosed to prevent identifying individuals.

**Table 2:** Distribution of Life Style Behaviors for Each Manufacturing Industry Group

Industry category	Brinkman Index^a^		Alcohol (g/day)	
	Median (IQR^b^25%:75%)		Median (IQR^b^25%:75%)	
	case	control	case	control
All	0 (0:660)	0 (0:125)	0 (0:45.0)	0 (0:0)
Food	0 (0:350)	0 (0:0)	0 (0:35.0)	0 (0:0)
Beverages, tobacco and feed	0 (0:720)	0 (0:60)	0 (0:72.0)	0 (0:0)
Textile mill products	0 (0:440)	0 (0:0)	0 (0:44.0)	0 (0:0)
Clothes and other textiles	0 (0:0)	0 (0:0)	0 (0:0)	0 (0:0)
Lumber and wood products, except furniture	0 (0:740)	0 (0:150)	0 (0:74.0)	0 (0:0)
Furniture and fixtures	0 (0:700)	0 (0:150)	0 (0:70.0)	0 (0:0)
Pulp, paper and paper products	0 (0:690)	0 (0:200)	0 (0:69.0)	0 (0:40.0)
Printing and allied industries	0 (0:680)	0 (0:102)	0 (0:68.0)	0 (0:4.0)
Chemicals, and chemical and allied products	0 (0:750)	0 (0:240)	0 (0:75.0)	0 (0:80.0)
Petroleum and coal products	285 (0:740)	0 (0:250)	28.5 (0:74.0)	0 (0:11.5)
Plastic products, except otherwise classified	0 (0:600)	0 (0:220)	0 (0:60.0)	0 (0:60)
Rubber products	0 (0:640)	0 (0:0)	0 (0:64.0)	0 (0:0)
Leather tannning, leather products and fur	0 (0:440)	0 (0:0)	0 (0:44.0)	0 (0:0)
Ceramic, stone and clay products	0 (0:780)	0 (0:260)	0 (0:78.0)	0 (0:10.5)
Iron and steel	0 (0:720)	0 (0:135)	0 (0:72.0)	0 (0:0)
Non-ferrous metals and products	0 (0:740)	0 (0:350)	15.0 (0:74.0)	0 (0:24.0)
Fabricated metal products	15 (0:735)	0 (0:200)	0 (0:73.5)	0 (0:30.0)
General purpose machinery	0 (0:720)	0 (0:202)	0 (0:72.0)	0 (0:55.0)
Electrical machinery, equipment and supplies	0 (0:660)	0 (0:560)	0 (0:66.0)	0 (0:0)
Information and communication electronics	0 (0:540)	0 (0:0)	0 (0:54.0)	0 (0:0)
Electronic parts, devices and electronic circuits	0 (0:470)	0 (0:60)	0 (0:47.0)	0 (0:0)
Transportation equipment	0 (0:765)	0 (0:200)	0 (0:76.5)	0 (0:60.0)
Precision machinery	0 (0:600)	0 (0:210)	0 (0:60.0)	0 (0:50.0)
Miscellaneous manufacturing industries	0 (0:510)	0 (0:320)	0 (0:51.0)	0 (0:0)

^a^Brinkman Index, the number of cigerettes smoked per day multiplied by the number of years smoked; ^b^IQR, Interquartile range.

**Table 3:** Odds Ratio of each Manufacturing Industry Category to Cancer

Industrial category	Prostate	Breast	Kidney	Ureter	Bladder	Esophagus	Stomach	Liver	Pancreas	Colon	Lung
Food (reference)	1.00(ref)	1.00(ref)	1.00(ref)	1.00(ref)	1.00(ref)	1.00(ref)	1.00(ref)	1.00(ref)	1.00(ref)	1.00(ref)	1.00(ref)
	-	-	-	-	-	-	-	-	-	-	-
Beverages, tobacco and feed OR(95%CI)	1.27(0.87-1.86)	1.18(0.82-1.71)	1.07(0.61-1.91)	1.73(0.83-3.62)	1.01(0.71-1.43)	1.02(0.67-1.54)	0.95(0.75-1.20)	0.96(0.68-1.37)	1.12(0.76-1.65)	1.01(0.86-1.40)	0.80(0.59-1.09)
p-value	0.207	0.359	0.802	0.145	0.966	0.932	0.686	0.834	0.562	0.446	0.163
Textile mill products OR(95%CI)	1.10(0.78-1.57)	1.09(0.82-1.55)	1.52(0.98-2.35)	1.90(1.03-3.51)	1.03(0.77-1.39)	1.03(0.71-1.49)	0.98(0.81-1.19)	1.16(0.87-1.54)	1.05(0.76-1.45)	1.00(0.81-1.24)	1.04(0.82-1.14)
p-value	0.583	0.562	0.057	0.040*	0.827	0.873	0.803	0.300	0.779	0.978	0.765
Clothes and other textiles OR(95%CI)	1.22(0.83-1.79)	1.22(1.04-1.44)	1.21(0.81-1.80)	1.70(0.96-3.00)	1.04(0.79-1.36)	0.79(0.54-1.16)	1.02(0.87-1.19)	1.04(0.80-1.35)	0.98(0.75-1.29)	1.11(0.95-1.29)	1.06(0.86-1.29)
p-value	0.300	0.016*	0.355	0.067	0.801	0.237	0.803	0.766	0.884	0.191	0.596
Lumber and wood products, except furniture OR(95%CI)	0.69(0.51-0.93)	0.82(0.59-1.12)	0.92(0.62-1.38)	1.18(0.65-2.09)	0.69(0.54-0.89)	0.54(0.39-0.75)	0.73(0.62-0.86)	0.51(0.38-0.67)	0.67(0.49-0.91)	0.74(0.61-0.88)	0.83(0.68-1.02)
p-value	0.014*	0.220	0.698	0.581	0.005**	0.000**	0.000**	0.000**	0.010*	0.001**	0.072
Furniture and fixtures OR(95%CI)	1.16(0.82-1.64)	1.34(0.84-2.15)	1.42(0.87-2.32)	1.76(0.88-3.51)	0.94(0.68-1.31)	0.56(0.35-0.89)	0.92(0.73-1.15)	0.64(0.44-0.93)	0.61(0.38-0.97)	1.16(0.92-1.46)	0.89(0.67-1.19)
p-value	0.400	0.219	0.164	0.112	0.731	0.014*	0.454	0.018*	0.037*	0.223	0.437
Pulp, paper and paper products OR(95%CI)	1.19(0.87-1.62)	1.05(0.76-1.44)	1.23(0.79-1.89)	0.55(0.24-1.27)	0.93(0.69-1.21)	0.75(0.53-1.06)	0.97(0.81-1.16)	0.82(0.61-1.09)	0.83(0.59-1.16)	1.05(0.86-1.27)	0.89(0.70-1.12)
p-value	0.266	0.772	0.355	0.162	0.524	0.107	0.724	0.182	0.290	0.639	0.312
Printing and allied industries OR(95%CI)	1.16(0.85-1.58)	1.30(0.99-1.69)	1.63(1.09-2.43)	2.01(1.15-3.52)	1.41(1.09-1.81)	1.26(0.92-1.71)	1.03(0.86-1.22)	1.03(0.78-1.36)	1.16(0.85-1.57)	1.37(1.15-1.64)	1.00(0.79-1.25)
p-value	0.353	0.053	0.016*	0.014*	0.008**	0.148	0.766	0.838	0.351	0.000**	0.997
Chemicals, and chemical and allied products OR(95%CI)	1.26(1.01-1.57)	1.26(1.01-1.58)	1.40(1.03-1.91)	0.97(0.59-1.58)	1.03(0.85-1.25)	0.67(0.52-0.87)	1.06(0.93-1.19)	0.87(0.71-1.06)	0.87(0.69-1.09)	1.13(0.98-1.29)	1.07(0.91-1.25)
p-value	0.040*	0.04*	0.030*	0.909	0.795	0.002**	0.398	0.174	0.234	0.086	0.411
Petroleum and coal products OR(95%CI)	1.83(1.16-2.89)	1.14(0.48-2.72)	1.94(1.02-3.69)	1.01(0.29-3.45)	1.58(1.04-2.41)	1.15(0.67-1.98)	1.47(1.09-1.98)	1.07(0.66-1.73)	1.16(0.65-2.05)	1.56(1.14-2.16)	1.51(1.04-2.18)
p-value	0.009**	0.770	0.045*	0.982	0.032*	0.605	0.011**	0.787	0.612	0.006**	0.029**
Plastic products, except otherwise classified OR(95%CI)	0.76(0.48-1.18)	1.37(0.99-1.88)	1.41(0.85-2.35)	0.77(0.29-2.00)	0.79(0.54-1.15)	1.04(0.69-1.57)	0.92(0.74-1.16)	0.67(0.45-1.00)	0.75(0.48-1.17)	1.09(0.87-1.39)	1.07(0.81-1.41)
p-value	0.218	0.050	0.189	0.586	0.216	0.835	0.485	0.054	0.204	0.446	0.623
Rubber products OR(95%CI)	0.92(0.52-1.63)	1.33(0.79-2.22)	1.74(0.89-3.39)	2.82(1.19-6.70)	1.33(0.86-2.06)	1.02(0.56-1.83)	1.11(0.81-1.52)	1.27(0.81-1.99)	1.19(0.69-2.04)	1.08(0.76-1.52)	0.94(0.62-1.42)
p-value	0.779	0.278	0.102	0.018*	0.196	0.949	0.503	0.294	0.535	0.680	0.767
Leather tannning, leather products and fur OR(95%CI)	1.81(0.56-5.85)	1.38(0.64-2.95)	1.99(0.58-6.81)	2.41(0.30-19.28)	1.95(0.79-4.81)	0.36(0.05-2.89)	1.89(1.13-3.17)	2.36(1.15-4.83)	2.85(1.26-6.47)	1.45(0.77-2.80)	2.00(1.01-3.99)
p-value	0.319	0.406	0.274	0.407	0.143	0.341	0.015*	0.019**	0.012**	0.243	0.028*
Ceramic, stone and clay products OR(95%CI)	0.87(0.66-1.13)	0.95(0.72-1.25)	0.93(0.64-1.36)	1.35(0.80-2.26)	1.02(0.82-1.26)	0.65(0.48-0.86)	0.94(0.82-1.08)	0.97(0.78-1.21)	0.78(0.59-1.02)	0.95(0.82-1.11)	1.26(1.06-1.49)
p-value	0.286	0.704	0.720	0.260	0.875	0.003**	0.400	0.771	0.075	0.538	0.008**
Iron and steel OR(95%CI)	1.19(0.95-1.47)	1.11(0.84-1.47)	1.15(0.84-1.56)	1.30(0.82-2.08)	1.08(0.89-1.30)	0.71(0.56-0.91)	1.03(0.91-1.16)	0.94(0.79-1.14)	0.98(0.79-1.23)	1.07(0.96-1.43)	1.04(0.89-1.21)
p-value	0.126	0.473	0.387	0.266	0.423	0.006**	0.213	0.540	0.898	0.294	0.615
Non-ferrous metals and products OR(95%CI)	1.18(0.87-1.61)	1.95(1.29-2.92)	1.18(0.89-2.14)	1.25(0.64-2.43)	1.28(0.98-1.67)	0.88(0.62-1.24)	1.12(0.94-1.35)	0.89(0.66-1.21)	1.15(0.83-1.59)	1.17(0.96-1.43)	1.18(0.91-1.43)
p-value	0.288	0.001**	0.149	0.520	0.063	0.459	0.213	0.488	0.410	0.110	0.267
Fabricated metal products OR(95%CI)	0.76(0.61-0.94)	0.81(0.67-0.98)	0.83(0.61-1.13)	0.78(0.49-1.24)	0.74(0.62-0.89)	0.72(0.57-0.90)	0.91(0.81-1.02)	0.77(0.63-0.92)	0.78(0.63-0.96)	0.95(0.85-1.08)	0.83(0.71-0.96)
p-value	0.013*	0.030*	0.236	0.290	0.002**	0.004**	0.096	0.005**	0.017*	0.439	0.011*
General-purpose mechinery OR(95%CI)	1.04(0.83-1.29)	1.14(0.92-1.41)	1.34(0.99-1.80)	1.29(0.81-2.02)	1.04(0.86-1.25)	0.64(0.49-0.81)	0.99(0.88-1.12)	0.86(0.71-1.04)	0.84(0.67-1.04)	1.10(0.97-1.25)	0.97(0.83-1.13)
Industrial category	Prostate	Breast	Kidney	Ureter	Bladder	Esophagus	Stomach	Liver	Pancreas	Colon	Lung
p-value	0.726	0.237	0.055	0.286	0.675	0.000**	0.953	0.135	0.120	0.126	0.709
Electrical machinery, equipment and supplies OR(95%CI)	1.60(1.19-2.16)	1.27(0.98-1.63)	2.49(1.75-3.55)	2.09(1.18-3.70)	1.46(1.13-1.88)	0.98(0.70-1.38)	1.37(1.16-1.61)	1.00(0.75-1.33)	1.15(0.85-1.57)	1.67(1.41-1.97)	1.56(1.27-1.92)
p-value	0.002**	0.069	0.000**	0.011*	0.003**	0.917	0.000**	0.991	0.368	0.000**	0.000**
Information and communication electronics OR(95%CI)	1.81(1.27-2.58)	1.48(1.10-1.98)	2.69(1.77-4.11)	2.14(1.02-4.45)	1.69(1.23-2.32)	1.11(0.73-1.69)	1.21(0.98-1.49)	0.87(0.59-1.27)	1.79(1.27-2.54)	1.46(1.78-1.81)	1.60(1.24-2.07)
p-value	0.001**	0.009**	0.000**	0.043*	0.001**	0.616	0.082	0.467	0.001**	0.001**	0.000**
Electronic parts, devices and electronic circuits OR(95%CI)	1.06(0.62-1.58)	1.13(0.88-1.45)	2.07(1.37-3.13)	1.75(0.86-3.53)	1.26(0.93-1.72)	0.59(0.36-0.95)	1.15(0.95-1.39)	0.83(0.58-1.18)	1.39(0.99-1.93)	1.43(1.18-1.73)	1.13(0.88-1.46)
p-value	0.765	0.329	0.001**	0.120	0.136	0.030*	0.139	0.290	0.051	0.001**	0.344
Transportation equipment OR(95%CI)	0.89(0.71-1.11)	0.99(0.78-1.27)	1.03(0.75-1.41)	1.41(0.89-2.22)	1.03(0.85-1.25)	0.61(0.47-0.78)	0.94(0.83-1.05)	0.85(0.70-1.60)	0.91(0.73-1.34)	0.97(0.85-1.11)	1.11(0.95-1.29)
p-value	0.302	0.961	0.873	0.140	0.746	0.000**	0.295	0.627	0.398	0.644	0.174
Precision machinery OR(95%CI)	0.92(0.58-1.44)	1.52(1.04-2.21)	1.16(0.62-2.17)	0.21(0.03-1.59)	1.11(0.76-1.62)	0.75(0.45-1.25)	1.14(0.89-1.46)	1.09(0.75-1.60)	1.00(0.63-1.60)	1.30(1.01-1.69)	0.98(0.70-1.36)
p-value	0.703	0.961	0.652	0.132	0.593	0.269	0.314	0.627	0.987	0.043*	0.894
Miscellaneous manufacturing industries OR(95%CI)	0.78(0.53-1.15)	1.20(0.92-1.58)	1.17(0.73-1.87)	1.57(0.82-2.99)	1.06(0.79-1.42)	0.63(0.41-0.95)	1.13(0.94-1.35)	0.70(0.51-0.98)	0.65(0.45-0.96)	1.15(0.94-1.39)	0.87(0.68-1.12)
p-value	0.209	0.185	0.516	0.176	0.686	0.031**	0.199	0.039*	0.031*	0.177	0.299

Odds ratios for a total of 40,370 cases were estimated by logistic regression. The model included age, sex, period of admission, admission hospital, smoking (Brinkman Index), and consumption of alcohol as covariates. p-values <0.01** or <0.05* were considered to be statistically significant.
